# Gut Microbiome 16S rRNA Gene Amplicon Taxonomic Profiling of Hospitalized Moroccan COVID-19 Patients

**DOI:** 10.1128/mra.00256-22

**Published:** 2022-06-08

**Authors:** Sofia Sehli, Abdellah Idrissi Azami, Nouzha Dini, Nihal Habib, Bouchra Chaouni, Salsabil Hamdi, Najib Al Idrissi, Saaid Amzazi, Imane Allali, Chakib Nejjari, Hassan Ghazal

**Affiliations:** a Laboratory of Genomics and Bioinformatics, Faculty of Pharmacy, Mohammed VI University of Health Sciences (UM6SS), Casablanca, Morocco; b Laboratory of Biotechnology and Plant Physiology, Center of Plant and Microbial Biotechnology, Biodiversity, and Environment, Department of Biology, Faculty of Sciences, University Mohammed V, Rabat, Morocco; c Environmental Health Laboratory, Department of Research, Institut Pasteur Du Maroc, Casablanca, Morocco; d Laboratory of Human Pathologies Biology, Department of Biology, Faculty of Sciences, University Mohammed V in Rabat, Rabat, Morocco; e Genomic Center of Human Pathologies, Faculty of Medicine and Pharmacy, University Mohammed V in Rabat, Rabat, Morocco; f National Center for Scientific and Technical Research, Rabat, Morocco; g Department of Surgery, Faculty of Medicine, Mohammed VI University of Health Sciences (UM6SS), Casablanca, Morocco; KU Leuven

## Abstract

We explored the gut microbiome composition in four Moroccan patients with coronavirus disease 2019 (COVID-19) during hospitalization and treatment, using 16S rRNA gene amplicon metataxonomic profiling, and compared it with that in four healthy severe acute respiratory syndrome coronavirus 2 (SARS-CoV-2)-free control subjects.

## ANNOUNCEMENT

To characterize the gut microbiome of hospitalized Moroccan patients with coronavirus disease 2019 (COVID-19) who were treated with azithromycin antibiotic and chloroquine, we analyzed the fecal microbiomes of four Moroccan COVID-19 patients at the Cheikh Khalifa International University Hospital in Casablanca, Morocco. We compared those gut microbiomes to those of four control subjects who were never in contact with severe acute respiratory syndrome coronavirus 2 (SARS-CoV-2) and thus were not hospitalized and not under treatment. The COVID-19 patient cohort had a male/female ratio of 1:3 and an age range of 30 to 57 years, while the control cohort had a male/female ratio of 2:2 and an age range of 32 to 63 years. The COVID-19 treatment protocol includes chloroquine (Nivaquine; Sanofi Morocco) at 500 mg twice per day for 7 days and azithromycin at 500 mg on day 1 and then 250 mg on days 2 to 7.

The four COVID-19 patients were hospitalized in the intensive care unit for 2 weeks following a positive diagnosis in April 2020. The first case of SARS-CoV-2 infection in Morocco was detected in March 2020 (1). Fecal samples (100 mg) were taken 10 days following the start of treatment, in Sarstedt tubes containing 2.5 mL of RNA*later* solution. The samples from the four control subjects were taken during the second half of 2019. Approval for sequencing of fecal microbiomes from human subjects was obtained from the local ethics committee: Ethics Committee for Biomedical Research (CERB) and Mohammed VI University of Health Sciences (UM6SS) (approval number: CERB/UM6SS/06/21). A QIAamp DNA stool minikit (Qiagen, Germany) was used to extract DNA. The hypervariable V3 to V4 region of the 16S rRNA gene was amplified as described by Klindworth et al. (2) using the primers S-d-Bact-0341-b-S-17 (5′-CCTACGGGNGGCWGCAG-3′) and S-d-Bact-0785-a-A-21 (5′-GACTACHVGGGTATCTAATCC-3′). The PCR conditions were as follows: initial denaturation at 95°C for 5 min, followed by 25 cycles of denaturation at 95°C for 40 s, annealing at 55°C for 2 min, and extension at 72°C for 1 min, with a final extension step at 72°C for 7 min. The Nextera XT index kit (Illumina, USA) was used to index PCR products, attaching dual indices and Illumina sequencing adapters. The Illumina MiSeq sequencer generated paired-end reads with an average size of 300 bases and an average number of reads per sample of 137,000.

The DADA2 (v.1.16) (3) pipeline was utilized for 16S rRNA gene amplicon sequence data bioinformatic analysis. Amplicon sequence variants (ASVs) were aligned to create a phylogeny using DECIPHER (4). Phangorn (5) was used to build a phylogenetic tree. Taxonomic assignment was determined by mapping the ASV table to the SILVA database (6) using DADA2. Phyloseq (7) was utilized to measure alpha and beta diversities. To characterize the different taxonomic compositions between SARS-CoV-2-free subjects and COVID-19 patients, we used DESeq2 (8). Plots were created using ggplot2 in R.

**FIG 1:**
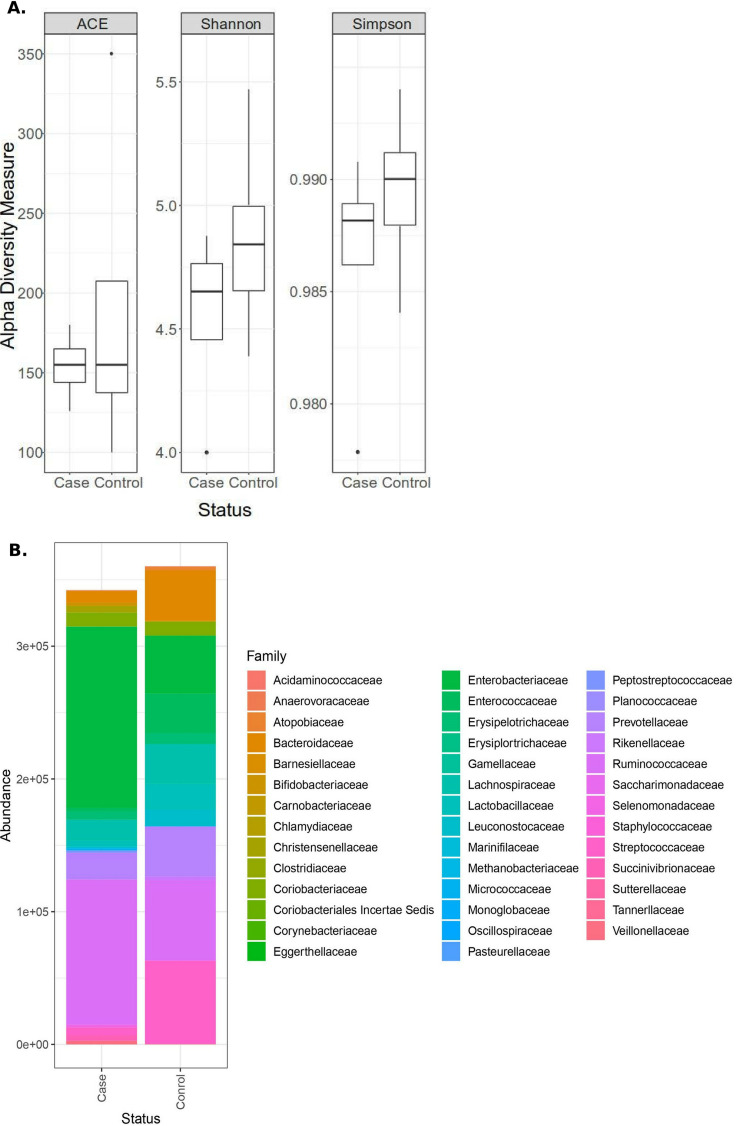
(A) Alpha diversity measures, including the average Shannon-Wiener (alpha) diversity index, abundance-based coverage estimator (ACE) index, and Simpson index, for 16S rRNA gene amplicons from stool samples from four COVID-19 patients (cases) and four control subjects, as represented in the box plots; each box represents quartiles, the horizontal lines within the boxes represent the medians, and the vertical lines outside the boxes are the outliers. (B) Bar plot depicting the microbial abundance at the family level within the control and COVID-19 (case) samples.

Our 16S rRNA gene metataxonomic profiling method revealed microbiome composition and richness changes in hospitalized COVID-19 patients, whose gut microbiomes were enriched with opportunistic pathogens such as Escherichia/*Shigella* (range, 52% to 63%) and depleted of beneficial commensals such as *Bacteroidetes* (0% to 20%). *Proteobacteria* (53% to 83%) was the most dominant phylum in the COVID-19 patient group, followed by *Firmicutes* (21% to 35%), *Bacteroidetes* (5% to 8.2%), and *Actinobacteria* (3.4% to 5.2%). In the control group, *Firmicutes* was the most abundant phylum (43% to 68%), followed by *Bacteroidetes* (32% to 44%), and *Proteobacteria* and *Actinobacteria* had very low abundances (3.2% to 5% and 3.5% to 5.4%, respectively). Interestingly, six genera were present only in the COVID-19 samples, namely, *Lachnospiraceae* NK3A20 group; *Succcinivibro*, a marker of human diarrheal feces (9); *Libanicoccus* (10); NK4A214 group (11); *Citrobacter* (12); and *Rothia*, which is likely involved in pneumonia (13) (Fig. 1). These findings support the expected impact of antibiotic treatment on the microbiome diversity of COVID-19 patients but point toward potential specific gut microbiota-SARS-CoV-2 interactions. A large cohort is needed to generalize these results.

### Data availability.

The raw sequence data from this project have been deposited under the BioProject accession number PRJNA728736. The Sequence Read Archive (SRA) accession numbers for the raw sequence data for the COVID-19 patients are as follows: SRX10859493, SRX10859492, SRX10859491, and SRX10859490. The SRA accession numbers for the raw sequence data for the control subjects are as follows: SRX10859497, SRX10859496, SRX10859495, and SRX10859494.
